# Can Sleep Parameters Predict Upcoming Mood Episodes in Bipolar Disorder?

**DOI:** 10.1111/bdi.70054

**Published:** 2025-08-22

**Authors:** Andrea Ulrichsen, Esther Mühlbauer, Lisa‐Marie Hartnagel, Emanuel Severus, Anthony Cleare, Sameer Jauhar, Michael Bauer, Ulrich Ebner‐Priemer

**Affiliations:** ^1^ Department of Psychiatry and Psychotherapy, University Hospital Dresden University of Technology Dresden Germany; ^2^ King's College London Institute of Psychiatry, Psychology and Neuroscience London UK; ^3^ Mental mHealth Lab, Institute of Sports and Sport Sciences Karlsruhe Institute of Technology Karlsruhe Germany; ^4^ Department of Psychiatry and Psychotherapy, Medical Faculty Mannheim, Central Institute of Mental Health University of Heidelberg Mannheim Germany; ^5^ German Centre for Mental Health (DZPG), Partner Site Mannheim Mannheim Germany

**Keywords:** bipolar disorder, depression, early warning signs, mania, mood prediction, sleep

## Abstract

**Background:**

Bipolar disorder (BD) is a recurrent disorder, characterised by episodes of (hypo)mania, depression and euthymia with variation in mood, cognition and sleep. Many patients identify changes in sleep before an episode; using daily sleep logs could help identify these changes. Such early warning signs can be a valuable tool for patients and clinicians alike in predicting and preparing for changes in mood.

**Methods:**

In the BipoSense study, we followed patients with BD, who were in remission at the start of the study, daily for 1 year. Patients reported for each hour if they were awake or asleep through an app and received fortnightly clinical assessments of bipolar symptoms. We used statistical analyses applying person‐centred data in multilevel logit models to investigate if sleep patterns could differentiate between the period before an episode (prodromal stage) and euthymia, looking at both mean changes and variability of sleep. Bonferroni‐Holm corrections were applied to avoid inflation of type I errors from multiple testing.

**Results:**

Twenty‐nine participants were included (mean age 44.0 years [SD = 11.9], female 55% and BD‐I 59%). Waking up later was associated with prodromal depression and was the only significant finding for prodromal mood episodes. Greater variability of sleep duration, total time spent in bed and time waking up were associated with prodromal depression; less variability of time falling asleep and time waking up were linked with prodromal (hypo)mania.

**Conclusion:**

Using self‐assessed sleep changes and especially variability can be potential tools in helping patients identify early warning signs of mood recurrence; however, these analyses were explorative and further investigations are warranted.

## 1 Introduction

People with Bipolar Disorder (BD) fluctuate between episodes of euthymia, mania or hypomania and depression, and their lives are characterised by substantial loss of both psychosocial functioning and up to 20 years of life [1]. Recurrence of episodes is common, and prevention of these is the main goal of treatment, as well as improvement of related disturbances, for example, sleep [1]. A systematic review of 11 randomised controlled trials (RCTs) found that focusing on early warning signs (EWS) of recurrence was beneficial for both the time to recurrence and the percentage of people hospitalised for their BD symptoms [2]. Identifying potential EWS can therefore be highly beneficial for patients. To this end, ecological momentary assessment (EMA) (i.e., daily data collection) is ideally suited to gather data from patients, as it avoids retrospectively collecting information and can detect daily changes, which could be relevant [3].

Clinically, changes in sleep are used to diagnose both mania and depression [4]. However, sleep disturbances are also commonly found in euthymia. A meta‐analysis of 21 studies found that compared to healthy controls (HC), euthymic BD patients sleep more and spend more time in bed, go to bed later and wake up more often during the night [5]. Although most of the included studies were based on data of less than 1 week's observations, sleep disturbance is consistently found in all phases of BD, and changes in sleep are commonly identified by patients as a prodrome to particularly a manic episode [6]. This is further supported by longitudinal studies [7, 8, 9] that recorded daily self‐assessed sleep and mood for several months. They found an inverse relationship between sleep variables and next day mood, particularly sleep duration [8] and sleep duration variability [9], where sleeping more was followed by an increase in depressive symptoms, and vice versa for mania. However, even though longitudinal studies are key in differentiating sleep patterns on a within‐subject level, those studies are often limited by the small number of incipient mood episodes [10].

A meta‐analysis from 2010 compared self‐rated to clinician‐rated mood in BD and found significant differences [11], highlighting the importance of reliable mood ratings. To the best of our knowledge, there have not to date been conducted any longitudinal studies investigating clinician‐rated changes in mood symptoms as well as differentiating between prodromal days and episode days. Furthermore, it has not been investigated whether sleep variables can be used to differentiate between these. The BipoSense study, by conducting frequent (every 2 weeks) and thorough expert‐rated mood assessments for 1 year, alongside daily sleep data collection, bridges this gap in the literature.

### 1.1 Aims and Objectives

This study aimed to investigate whether daily recorded sleep data over 1 year could be used to predict upcoming depressive or manic episodes in patients with BD. The primary objective was to investigate if sleep variables, for example, sleep duration or time falling asleep, can differentiate prodromal or episode phases from euthymia. The secondary objective was to investigate if statistical parameters of dynamic processes, for example, standard deviation (SD), may be useful in the prediction of prodromal or episode phases of BD. All the analyses were exploratory and were not preregistered.

## 2 Patients and Methods

### 2.1 Study Protocol

This is a secondary data analysis of the data collected for the BipoSense study [12]. The study was approved by the local ethics committee at the medical faculty of the Technical University of Dresden (EK‐Nr.: 26012014) and upheld the Declaration of Helsinki. The study followed participants with BD over 12 months, performing clinical interviews fortnightly, collecting daily e‐diary ratings on mood and sleep, as well as continuous digital phenotyping. This paper will focus on the fortnightly expert‐rated clinical mood ratings and self‐reported daily sleep data.

### 2.2 Procedures

Participants were recruited through a specialised outpatient clinic at University Hospital Dresden, Germany, and through articles in both print and online media. Inclusion criteria for participation were a diagnosis of BD Type I or II, in remission at the time of enrolment, defined as YMRS (Young Mania Rating Scale) [13] score ≤ 12 and MADRS (Montgomery and Asberg Depression Rating Scale) [14] score ≤ 12. A full list of inclusion criteria is previously published [12].

Eligible and willing participants were invited to participate. All participants gave written informed consent to participate before any study collection took place. Psychopathological assessments (see below) were carried out at the first visit, and every 2 weeks after this date, by a trained clinical psychologist either in person or over the phone. Furthermore, daily sleep data were collected with the MovisensXS app (movisens GmbH, Karlsruhe, Germany). Items were adapted from ChronoRecord [15], a validated computer‐based tracking system of mood, medication and sleep. Participants were prompted every evening at multiple times by the app to fill out their end‐of‐day e‐diary. The day was stored as missing if they did not respond. Participants were compensated with 35 Euros/month and smartphone data packages or a study phone.

### 2.3 Measures

#### 2.3.1 Clinical Interviews

Clinically trained psychologists assessed each participant every 2 weeks for 1 year for any affective episode in the last 2 weeks, using the SCID‐I section A according to DSM‐5 [16]. The German version of the YMRS, the Bech‐Rafaelsen Mania Rating Scale (BRMRS) [17] and the MADRS were used to assess the severity of symptoms. We made one change from the DSM‐5 diagnostic criteria to optimise our data: two episodes only had to be separated by a 14‐day period of euthymia to be distinct episodes.

#### 2.3.2 E‐Diary of Sleep

Daily end‐of‐day questions were sent through the movisensXS app. The sleep items consisted of 24 1‐h blocks, which could be marked as asleep, awake in bed or awake, resulting in hourly information on sleep/wake behaviour. Specific sleep indices (duration of sleep and sleeplessness, and time falling asleep and waking up) were calculated from this.

### 2.4 Data Preparation

IBM SPSS Statistics (version 29.0.1.0 [171]) was used for data preparation and analysis.

#### 2.4.1 Sleep Data

The four sleep variables used were ‘sleep duration (hours)’, ‘sleepless in bed (hours)’, ‘time waking up’ and ‘time falling asleep’. In order to see the person‐specific values, instead of group values, we calculated person‐centred variables allowing us to see how the participant's sleep was different to their own sleep averages (see Data S1 for SPSS syntax). To identify the hour the participant went to sleep, we re‐coded any hour where a participant went from either awake to asleep, or sleepless in bed to asleep, as the hour the participant fell asleep. Acknowledging the potential of waking up and falling asleep again multiple times, we used the earliest falling asleep hour as the time the participant fell asleep. To prevent using day‐time naps as the start time of falling asleep, we only used the hours between 20:00 and 05:00 for this calculation. Days where participants did not sleep in this time frame were not included.

#### 2.4.2 Expert Rated Episode Phase

**FIGURE 1:**
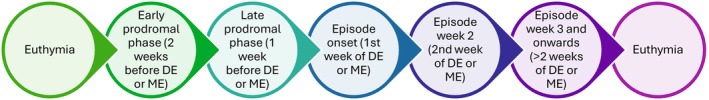
Episode phase as the analysis outcomes. Hypomania and mania are combined into one mood category, (hypo)mania. Euthymia status is defined as any timepoint not otherwise specified as an episode phase. DE = depressive episode, ME = (hypo)manic episode.

In order to investigate whether sleep changes associated with BD episodes can be detected before a new episode begins, the variable ‘Episode Phase’ was created and is our main outcome. This variable categorises ‘early prodromal’ phase 14–8 days before episode onset and ‘late prodromal’ phase 7‐1 days before onset, for both depressive episodes (DE) and (hypo)manic episodes (ME). It also differentiates Days 1–7, 8–14 and 15+ days after episode start, with the latter group continuing until the episode has ended. All other days are categorised as euthymic. As we did not have enough data to run separate analyses of hypomanic and manic episodes, these were combined into one category, called (hypo)mania episode (ME). In total, there were five different episode phases for each episode category (depression and (hypo)mania), as well as one for euthymia, making a total of 11 separate episode phases. See Figure 1.

#### 2.4.3 Missing Data

If the fortnightly interviews were not exactly scheduled with a 14‐day interval, but 1 or 2 days later (i.e., after 15 or 16 days), missing data between the two classified 14‐day intervals occurred. A few cases (*n* = 4) had 2–7 missing days before the coded episode phase (new episode). These were categorised as euthymic. Furthermore, four patients started with a depressive episode, as diagnosed at the first clinical assessment, 14 days after beginning the study. Here no prodromal days are recorded.

#### 2.4.4 Analyses and Statistical Models

The primary aim of the analyses was to investigate if sleep parameters (as statistical predictor) can differentiate between euthymic days and the various episode phases (outcome), or in other words, if changes in sleep can statistically predict mood episode. To this end, we ran general linear mixed multilevel logit models for each episode phase with each of the four sleep variables (within‐person mean values), the predictors, as fixed effects. See Data S2 for the SPSS syntax.

To investigate how the within‐person variability of the sleep variables was associated with the episode phase, we also calculated the standard deviation (SD) for each sleep variable. To do this, we built the SD for each sleep index for the previous 7 days (starting at Day 7 of the 365 recorded days). The SD sleep indices were afterwards within‐person centred, and we used the same multilevel models for each episode phase as were used for the sleep variable means. Similarly to how we found the SD, we investigated the instability using the sum of squared deviations (SSD). To avoid type I error inflation when doing multiple comparisons, the Bonferroni‐Holm correction was applied to all our results. The Holm method is a Bonferroni‐based model, which controls for family‐wise errors [18]. We have included both correction for 5% significance, as well as 1%, called high significance. The full strategy, as well as the results of the correction, are included in Tables 2 and 3, and Data S3. To show transparency, we have included both results below (before and after correction).

**FIGURE 2:**
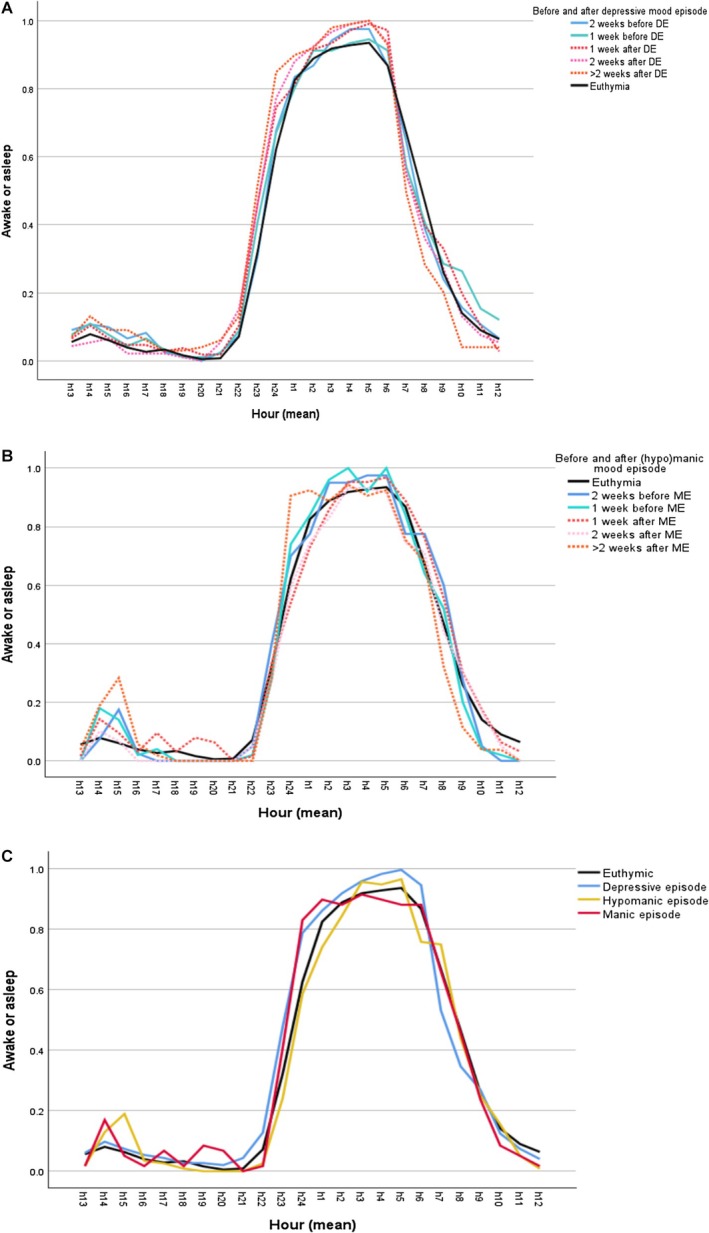
(A) Sleep patterns across depressive episode phase. (B) Sleep patterns across (hypo)manic episode phase. (C) Sleep patterns across mood episodes. (A–C) Time in bed (asleep or awake) differences for depressive Episode phase (A), (hypo)manic episode phase (B), and all mood episodes (C). The graphs show the various hours (h) of the day patients on average were either awake or asleep (0 = awake, 1 = asleep).

For the visual presentation of sleep patterns, we created a simple line graph of means for each hour for asleep or sleepless in bed, where 1 illustrates sleep and 0 awake, for each episode phase. Furthermore, we used only data from participants who had experienced ME to create the manic episode phase graphs, and vice versa for DE, which allowed the euthymia data to represent only euthymic phases for those who also experienced ME/DE. See Figure 2A–C.

## 3 Results

### 3.1 Participants

In total, 53 patients were screened for inclusion criteria, and 31 patients were included. Two participants were excluded due to technical difficulties, and the final study sample was 29 participants. The average age of the sample was 44.0 years (SD = 11.9, range: 25–70); 55% were female (16/13), and 59% had BD‐I diagnosis (17 BD‐I/12 BD‐II). Mean lifetime affective episodes were for depression 7.1 (SD = 5.6), hypomania 3.0 (SD = 3.8), and mania 2.8 (SD = 3.5), and participants had been admitted to hospital for BD on average 3.6 times (SD = 3.7, range: 0–15). In total, participants were followed over 10,587 study days (mean per participant = 365, range: 308–398 days). There was excellent compliance: 97% (*N* = 726) of the scheduled fortnightly clinical interviews were completed, and 89% (*N* = 9433) of the daily e‐diaries were filled out. On average per night, participants slept 7.9 h (SD = 1.3), were sleepless in bed for 0.8 h (SD = 0.9), woke up at 8:39 (SD = 1 h 44 min) and went to sleep at 00:21 (SD = 1 h 3 min). See Table 1 for all details.

**TABLE 1:** Participant characteristics.

	*N*	Mean (overall)	Minimum (participant means)	Maximum (participant means)	SD
Age	29	44.0	25.00	70.00	11.90
Gender	29	Female: 55.2% (16 = female, 13 = male)			
BD diagnosis	29	BD type 1 = 58.6% (BD‐I = 17, BD‐II = 12)			
Lifetime depressive episode	29	7.1	2	30	5.6
Lifetime hypomanic episode	29	3.0	0	15	3.8
Lifetime manic episode	29	2.8	0	10	3.5
Lifetime hospital admissions (BD)	29	3.6	0	15	3.7
Hours awake	29	15.4	12.9	17.2	1.0
Sleepless in bed (h)	29	0.8	0.1	3.6	0.9
Sleep duration (h)	29	7.9	4.7	10.3	1.3
Time waking up (time)	29	8:39	03:19	14:25	1 h 44 min
Time falling asleep (time)	29	00:21	22:23	3:58	1 h 3 min

Abbreviations: BD, bipolar disorder; *N*, number of participants; SD, standard deviation.

### 3.2 Sleep and Mood

#### 3.2.1 Episode Phase and Sleep

As expected, the average sleep duration (hours) differed between the episode phases on a descriptive level. Sleep duration (hours) ranged for the depressive episode phase between 7.71 (SD = 1.65) 2 weeks after episode onset to 7.93 (SD = 1.71) 2 weeks before episode onset, and for the (hypo)manic episode phase from 7.02 (SD = 1.93) 3 weeks after episode onset to 7.52 (SD = 1.24) 1 week before episode onset. Mean sleep duration (hours) for euthymia was 7.87 h (SD = 1.24). Lowest sleep hours were 7.02 h (SD = 1.93) > 2 weeks of ME, and longest sleep hours were 7.93 h (SD = 1.71) early prodromal DE. Although most differences were in line with clinical assumptions [4], the differences were—descriptively—rather small. See Data S4 for all results.

Figure 2A,B portrait sleep patterns for each episode phase, showing whether participants were awake or asleep at a given hour. In general, 24 h‐sleep‐awake patterns across episode phases were surprisingly similar; but the graphs still indicate less time awake at night during depression phases and more daytime sleeping (naps) during (hypo)mania. This is further supported by Figure 2C, which shows the sleep patterns of the 4 mood states of BD (euthymia, depression, hypomania and mania).

#### 3.2.2 Sleep Data (Sum) Predicting Episode Phase, Multilevel Models

Our first aim was to investigate if our four sleep variables could predict the prodromal states using daily sleep values. A positive coefficient, for example, between ‘sleep duration (hours)’ and the episode phase late prodromal phase of DE, would denote that more hours asleep, compared to euthymia, is related to a higher probability of being in the late prodromal phase of DE and vice versa for negative coefficient. For ‘time falling asleep and waking up’, a positive coefficient indicates that falling asleep or waking up later statistically predicts a higher chance of being in the relevant mood phase compared to euthymia. The multilevel model results are displayed in Table 2. Overall, just a few results reached significant values.

**TABLE 2:** Predicting episode phase from sleep data (mean).

	Episode phase	Days	Coefficient	SE	*p*
Sleep duration (h)	Euthymia	7117–7158			
	2 weeks before DE	167	0.089	0.0532	0.095
	1 week before DE	151	0.04	0.0567	0.479
	1 week after DE	183	0.051	0.0532	0.961
	2 weeks after DE	167	0.01	0.056	0.859
	> 2 weeks after DE	174	0.026	0.0508	0.609
	2 weeks before ME	111	−0.016	0.0577	0.785
	1 week before ME	107	0.041	0.0617	0.502
	1 week after ME	132	0.004	0.0529	0.941
	2 weeks after ME	129	−0.083	0.052	0.109
	> 2 weeks after ME	94	−0.15	0.062	0.015
Sleepless in bed (h)	Euthymia	7158			
	2 weeks before DE	167	−0.11	0.0925	0.235
	1 week before DE	151	−0.038	0.0835	0.65
	1 week after DE	183	0.106	0.0582	0.068
	2 weeks after DE	167	0.11	0.0623	0.076
	> 2 weeks after DE	174	0.129	0.0567	0.023
	2 weeks before ME	111	0.05	0.0717	0.482
	1 week before ME	107	−0.21	0.0968	0.03
	1 week after ME	132	−0.106	0.0792	0.183
	2 weeks after ME	129	−0.187	0.0874	0.033
	> 2 weeks after ME	94	0.021	0.073	0.769
Time falling asleep	Euthymia	6817–6858			
	2 weeks before DE	164	0.01	0.0743	0.894
	1 week before DE	145	−0.009	0.0801	0.91
	1 week after DE	179	−0.196	0.0764	0.01
	2 weeks after DE	166	−0.151	0.0795	0.058
	> 2 weeks after DE	171	0.01	0.059	0.87
	2 weeks before ME	110	0.014	0.0798	0.861
	1 week before ME	106	−0.09	0.0826	0.277
	1 week after ME	132	0.039	0.0708	0.583
	2 weeks after ME	127	0.03	0.072	0.682
	> 2 weeks after ME	94	−0.098	0.0911	0.282
Time waking up	Euthymia	7083–7125			
	2 weeks before DE	167	−0.012	0.0546	0.825
	1 week before DE	147	0.112	0.0527	0.033
	1 week after DE	183	0.004	0.0525	0.935
	2 weeks after DE	166	−0.053	0.0594	0.372
	> 2 weeks after DE	173	−0.231	0.0518	< 0.001**
	2 weeks before ME	111	0.018	0.0645	0.782
	1 week before ME	105	−0.098	0.0697	0.158
	1 week after ME	132	−0.066	0.0591	0.263
	2 weeks after ME	129	−0.008	0.0587	0.889
	> 2 weeks after ME	94	−0.172	0.0824	0.037

*Note:* Results of multilevel models predicting likelihood of episode phase compared to euthymia based on sleep variables (within‐centred means). ‘Euthymia days’ is displayed as a range, as number of euthymia days to compare to varied.

Abbreviations: DE, depressive episode; ME, (hypo)mania episode; *p*, significance; SE = standard error.

** High significance after correction for Bonferroni‐Holm.

##### 3.2.2.1 Predicting Prodromal Phase of Depression

The multilevel logit models revealed no significant effects of ‘sleep duration (hours)’, ‘sleepless in bed (hours)’ or ‘time falling asleep’ as predictors for either early or late prodromal DE compared to euthymia. Only ‘time waking up’ models revealed a significant positive effect for late prodromal DE versus euthymia (*p* = 0.033), denoting that waking up later was related to a higher probability of an upcoming depressive episode. ‘Time waking up’ models revealed no significant effect for early prodromal DE versus euthymia (*p* = 0.825).

##### 3.2.2.2 Predicting Depressive Episode

Similar to the prodromal phases, our results show tendencies towards the expected effects, but the multilevel logit models did not reach significance for ‘sleep duration (hours)’ and ‘sleepless in bed (hours)’ as predictors of DE. ‘Time falling sleep’ models revealed a significant negative effect for 1st week of DE versus euthymia (*p* = 0.01), with 2nd week of DE effect nearly reaching significance (*p* = 0.058), denoting that falling asleep earlier is related to a higher probability of being in DE. ‘Time waking up’ models only showed significant negative effect > 2 weeks of DE versus euthymia (*p* < 0.001), denoting that waking up earlier was related to a higher probability of being depressed. There were no significant effects of ‘time waking up’ for earlier in the episode (*p* = 0.94 and *p* = 0.37, respectively 1st and 2nd week of DE).

##### 3.2.2.3 Predicting Prodromal Phase (Hypo)mania

The multilevel logit models did not reveal significant effects for ‘sleep duration (hours)’, ‘time falling asleep’ and ‘time waking up’, as predictors of prodromal phase to ME. The only significant effect was found for ‘sleepless in bed (hours)’ in late prodromal ME versus euthymia (*p* = 0.03). The effect was negative, that is, fewer hours of ‘sleeplessness in bed’ was related to a higher chance of being close to a ME.

##### 3.2.2.4 Predicting (Hypo)mania Episode

Our models appeared to be a little more successful in predicting (hypo)mania, however it was not consistent for all the individual episode phases. We found a negative effect of ‘sleep duration (hours)’ > 2 week of ME versus euthymia (*p* = 0.015; denoting that less sleep is related to a higher probability of being in a manic episode), but not for the 1st or 2nd week of ME (*p* = 0.94 and *p* = 0.11, respectively). A negative effect was found regarding ‘sleepless in bed (hours)’ 2nd week of ME versus euthymia (*p* = 0.03), signifying fewer hours of being sleepless at night is related to a higher chance of being in a manic episode, but not for 1st or > 2 week of ME (*p* = 0.18 and *p* = 0.77, respectively). The negative effect of ‘time waking up’ > 2 weeks of ME versus euthymia (*p* = 0.04), indicates that waking up earlier was related to a higher probability of being in a manic episode, but not for 1st or 2nd week of ME (*p* = 0.26 and *p* = 0.89, respectively). No effect was found for ‘time falling asleep’ as predictor of ME.

#### 3.2.3 Sleep Data (Standard Deviation) Predicting Episode Phase, Multilevel Models

Our second aim was to explore if dynamic patterns of sleep over the previous seven nights, such as the SD rather than the mean, could predict episode phase. The multilevel results are displayed in Table 3. Compared to the mean‐level results above, a more promising pattern for prediction emerged.

**TABLE 3:** Predicting episode phase from sleep data (standard deviation).

	Episode phase	Days	Coefficient	SE	*p*
SD of sleep duration (h)	Euthymia	7572–7628			
	2 weeks before DE	182	0.415	0.0996	0.001**
	1 week before DE	164	0.403	0.1075	0.001**
	1 week after DE	182	0.434	0.103	0.001**
	2 weeks after DE	191	0.156	0.1208	0.196
	> 2 weeks after DE	190	0.115	0.1182	0.332
	2 weeks before ME	117	0.138	0.0868	0.111
	1 week before ME	104	−0.53	0.1975	0.007*
	1 week after ME	134	−0.068	0.1229	0.581
	2 weeks after ME	139	−0.013	0.1066	0.904
	> 2 weeks after ME	106	−0.256	0.1701	0.133
SD of sleepless in bed (h)	Euthymia	7572–7628			
	2 weeks before DE	182	−0.09	0.1394	0.518
	1 week before DE	164	0.172	0.1189	0.148
	1 week after DE	182	0.256	0.1107	0.021
	2 weeks after DE	191	0.278	0.1094	0.011
	> 2 weeks after DE	190	0.35	0.35	< 0.001**
	2 weeks before ME	117	0.169	0.1528	0.269
	1 week before ME	104	0.331	0.154	0.031
	1 week after ME	134	−0.605	0.1926	0.002*
	2 weeks after ME	139	−0.925	0.2131	< 0.001**
	> 2 weeks after ME	106	−0.303	0.1748	0.083
SD of time falling asleep	Euthymia	7376–7432			
	2 weeks before DE	182	−0.011	0.1831	0.951
	1 week before DE	163	−0.169	0.1987	0.394
	1 week after DE	182	0.159	0.1767	0.369
	2 weeks after DE	191	−0.122	0.1805	0.5
	> 2 weeks after DE	190	0.177	0.177	0.18
	2 weeks before ME	117	−0.029	0.1908	0.879
	1 week before ME	104	−0.715	0.2222	0.001**
	1 week after ME	134	−0.294	0.1909	0.124
	2 weeks after ME	139	−0.419	0.1884	0.026
	> 2 weeks after ME	106	−0.413	0.215	0.055
SD of time waking up	Euthymia	7566–7622			
	2 weeks before DE	182	−0.023	0.1252	0.857
	1 week before DE	164	0.647	0.1042	< 0.001**
	1 week after DE	182	0.527	0.1011	< 0.001**
	2 weeks after DE	191	0.23	0.1088	0.035
	> 2 weeks after DE	190	−0.048	0.105	0.646
	2 weeks before ME	117	−0.522	0.1803	0.004*
	1 week before ME	104	−0.592	0.1929	0.002*
	1 week after ME	134	−0.754	0.1745	< 0.001**
	2 weeks after ME	139	−0.123	0.1396	0.379
	> 2 weeks after ME	106	−0.575	0.1956	0.003*

*Note:* Results of multilevel models using within‐centred SD, predicting likelihood of episode phase compared to euthymia based on sleep variables SD. Euthymia days is displayed as a range, as number of euthymia days to compare to varied.

Abbreviations: DE, depressive episode; ME, (hypo)mania episode; *p*, significance; SD, standard deviation; SE = standard error.

* Significance after correction for Bonferroni‐Holm.

** High significance after correction for Bonferroni‐Holm.

##### 3.2.3.1 Predicting Prodromal Phases of Depression

As can be seen in Table 3, the multilevel logit models revealed positive significant effect of ‘SD of sleep duration (hours)’ as a predictor for prodromal depressive phases (early prodromal DE vs. euthymia: *p* = 0.001; late prodromal DE vs. euthymia: *p* = 0.001). The ‘SD of sleep duration (hours)’ was able to differentiate prodromal DE from euthymic days, indicating that more variability in the sleep duration was related to a higher probability of leading up to DE. In contrast, there were no significant effects of the predictors ‘SD of sleepless in bed (hours)’ and ‘SD of time falling asleep’. Furthermore, the multilevel logit models revealed a significant effect of ‘SD of time waking up’ as a predictor for late prodromal DE versus euthymia (*p* = 0.001), indicating more variability of wake‐up time the week before a depressive episode, but not for early prodromal DE versus euthymia (*p* = 0.857).

##### 3.2.3.2 Predicting Depressive Episode

We found significant effect of ‘SD of sleep duration (hours)’, ‘SD of sleepless in bed (hours)’ and ‘SD of time waking up’ as a predictor of 1st week of DE versus euthymia (*p* = 0.001, *p* = 0.021 and *p* < 0.001, respectively). All effects were positive denoting that more variability over the previous seven nights was related to a higher probability of being in DE. The models revealed ‘SD of sleepless in bed (hours)’ to be a particularly good variable in predicting any phase of DE, not only for 1st week, but for 2nd week and > 2 weeks as well (*p* = 0.011 and *p* < 0.001, respectively). In contrast, we did not find any significant effect of ‘SD of time falling asleep’ as predictor of DE (*p* = 0.37, *p* = 5, *p* = 0.18, respectively for 1st, 2nd or > 2 weeks).

##### 3.2.3.3 Predicting Prodromal Phases (Hypo)mania

Our results revealed that all sleep variables were good predictors of late prodromal ME: ‘SD of sleep duration (hours)’, *p* = 0.007; ‘SD of sleepless in bed (hours)’, *p* = 0.031; ‘SD of time falling asleep’, *p* = 0.001; ‘SD of time waking up’, *p* = 0.002. However, we only found effect of ‘SD of time waking up’ as predictor of early prodromal ME versus euthymia (*p* = 0.004). All significant results, apart from ‘SD of sleepless in bed (hours)’, had a negative coefficient, indicating less variability compared to euthymia increases probability of being in late prodromal ME.

##### 3.2.3.4 Predicting (Hypo)mania Episode

Our results revealed significant effects for three sleep variables (‘SD of total sleepless hours’, ‘SD of time waking up’ and ‘SD of time falling asleep’) in predicting ME in different weeks. In detail, we found a significant effect of ‘SD of sleepless in bed (hours)’ predicting 1st week of ME (*p* = 0.002) and 2nd week of ME (*p* < 0.001), but not > 2 weeks (*p* = 0.083). Furthermore, we found effect of ‘SD of time falling asleep’ 2nd week of ME versus euthymia (*p* = 0.026) and ‘SD of time waking up’ 1st week of ME vs. euthymia (*p* < 0.001) and > 2 weeks (*p* = 0.003). All coefficients were negative, denoting that lower variability was related to a higher chance of being in a ME. We did not find effect of ‘SD of sleep duration (hours)’ in predicting ME.

As SD covers variability but not instability, we additionally ran models with the SSD index. The SSD models revealed comparable findings to our SD models, indicating more between‐days instability of sleep variables in DE versus euthymia and less instability in ME versus euthymia, but have not been included here in full (see Data S5 for the results).

##### 3.2.3.5 Correcting for Multiple Testing (Bonferroni‐Holm Correction)

After applying the Bonferroni‐Holm correction, only one out of eight results remained significant of the mean sleep data, but 14 out of 19 of the SD sleep data results continued to be significant independent of the corrections; nine of these were highly significant. In particular, the results of SD of sleep duration and SD of time waking up remain significant (see Tables 2 and 3).

## 4 Discussion

This study explored the relationship between various sleep variables and mood in a comprehensive dataset of more than 10,000 days. Our main objective was to investigate if the weeks before a BD episode started or the first weeks of a new episode differed from the euthymic state on different sleep variables, and if sleep data could be used to predict a new mood episode. Given the multiple testing approach and risk of inflation of type I error, we applied Bonferroni‐Holm correction to all our results.

We first looked at the four different episode categories in BD: depression, hypomania, mania and euthymia, and at which hours participants on average would be asleep in each episode. These results are visually displayed in Figure 2A–C, where different sleep patterns appear to emerge. For depression, we observed going to sleep earlier in the evening and waking up earlier, similar to morning insomnia described in diagnostic manuals [4]. For mania and hypomania, the sleep patterns are more scattered, similar to what one might expect when having less need for sleep or sleeping at odd hours due to trouble falling asleep at night [4]. Although it was beyond the scope of this paper to calculate statistical differences between each hour for each episode category, the patterns of sleep in our data appear to be similar to clinical descriptions of sleep in BD in the DSM.

We then looked at how the different sleep variables could differentiate between episode phase and euthymia. We found little predictive value of person‐centred mean changes in sleep behaviour on episode phase, as only a few of the analyses were of significance. Overall, our results support that compared to euthymia, it is more likely that patients who wake up later are in late prodromal DE; those who fall asleep earlier are in the 1st week of DE; and those who spend more time sleepless in bed or wake up earlier are in > 2 weeks of DE. It is also more likely that those who spend less time sleepless in bed are in either late prodromal ME or 2nd week of ME, and those who sleep less or wake up earlier are in > 2 weeks of ME. Thus, waking up earlier, compared to euthymia, predicts both > 2 weeks of DE and ME. Sleeping more or experiencing insomnia is consistent with DSM criteria for depression, as is sleeping less and waking up early for mania [4]. Surprisingly, though, we did not find that greater or lesser sleep duration (hours) predicted prodromal phase or DE. The reason for this could be the mixed portrait of sleep described for depression in diagnostic manuals [4]: although in general for depression people are more tired and have less energy, some people sleep more, and some people sleep less due to insomnia. Combining sleep parameters with other passively tracked parameters might increase predictive power [19], as could using more sophisticated prediction models [20]. It is noteworthy that after correcting for multiple testing risk of errors, the only significant finding we have is earlier wake‐up being related to > 2 weeks of DE.

We had also expected to see sleep duration to be different in the weeks leading up to a manic episode, given sleep changes are commonly reported by patients as prodrome to mania [6]. This is also frequently reported in studies which have investigated daily changes in sleep and mood [8]. We did not see this in our data. An explanation for this could be the low number of manic episodes recorded, or the combining of mania and hypomania into one category. Another explanation could be our use of the weekly average of sleep—many studies have looked at daily changes, whereas we combined the 7 days into one category. The data may lose some nuance by doing this. It would be interesting in future studies to look at the day or days before an episode and investigate if these days carry more predictive value of an upcoming episode.

At last, we looked at variability in sleep to see if that could be used as a predictor of episode phase. Here, much more of our results reached statistical significance, indicating that variability in sleep holds greater predictive value than mean changes. All results largely remained significant after Bonferroni‐Holm correction. Overall, our results support that, compared to euthymia, it is more likely that patients who have more variability of sleep duration are in late prodromal or 1st week of DE and those who have less variability of sleep duration are in late prodromal ME; those who have more variability of sleepless hours are in 1st, 2nd or > 2 week of DE or in late prodromal ME, and those with less variability of sleepless hours are in 1st or 2nd week of mania; those who have less variability in the time they fall asleep are in late prodromal or 2nd week of mania; and finally, those who have more variability in the time they wake up are in late prodromal, 1st or 2nd week of depression, and those with less variability in the time they wake up are in early or late prodromal mania, 1st or > 2 week mania. For the majority of the results, more variability was overwhelmingly linked to the likelihood of prodromal DE or DE, whereas less variability in sleep patterns was linked to ME. Given the relative complexity of calculating SD, changes in variability are not something we would expect patients to self‐report easily. Instead, this could be uniquely measured and calculated by digital technology and alert appropriately of incipient episodes of BD.

In order to make our results more clinically relevant, all the analyses compared the various episode phase with person‐specific euthymia, allowing our results to reflect individual changes and variability in sleep, which are usually the only benchmark patients would have. However, as mentioned earlier, sleep variables in euthymia differ from healthy controls, and are not considered a healthy sleep [5]. It would be interesting to see future studies compare the various episode phase not to euthymia but to a general healthy sleep, to see if differences in prodromal states are more prominent then.

### 4.1 Limitations

Although this is a very comprehensive data set, with sleep/awake data for every hour of every day for a whole year, there are some limitations to the study design and methods used which are important to mention: First, patients were prompted to record their own sleep behaviour, and previous research has shown sleep duration to often be underestimated by BD patients [21]. However, sleep diaries are a validated tool in collecting sleep information and are easily accessible to patients during a study and as a tool for detecting EWS in their daily lives. Second, although the data set is impressive in size (10,587 study days), there were only 29 BD patients included. This comes across in the modest number of episodes recorded throughout the year, where in particular few manic episodes occurred. In order to combat this, we combined both mania and hypomania into one category; however, these are not exactly the same mood states, and differences between them in regard to sleep pattern could explain some of the inconclusive findings we had. Some of the nuances between mania and hypomania can visually be observed in Figure 2C; however, we did not calculate if the differences between mania and hypomania were significant due to the limited number of episode days recorded in our data. Furthermore, to increase the number of episodes recorded, we allowed new episodes to be recorded with only 2 weeks of euthymia in‐between, which is different from the DSM‐5 diagnostic criteria, where a much longer period of euthymia is needed to categorise it as a new episode [4]. Thirdly, in order to use sleep duration as a predictor, we averaged each day's sleep hours. However, by doing so we could miss out on nuances such as differences between sleeping uninterrupted throughout the night and having multiple wake‐ups, as well as differing between sleeping all hours at night or combining night sleep with day sleep (naps). These nuances could be explored further in future analyses. Similarly, investigating how self‐rated mood compares to clinical ratings could be undertaken. Furthermore, to avoid the potential bias when using self‐rated sleep, analyses could combine self‐reported sleep duration and sleepless in bed duration to provide a more robust variable [8]. Fourthly, we have not included data on medications participants may have taken throughout the study. This data were self‐report and difficult to verify, but of course could influence relapse rate. However, we believe there is value in looking at EWS in patients under treatment as usual, without differentiating between medication status, as this would closer replicate clinical settings. Finally, our approach is limited by the fact that the categorisation into early prodromal and late prodromal phase was based solely on a time criterion. Specifically, this means that some of the study participants may have already exhibited affective symptoms, albeit on a subsyndromal level, during these periods, while others were completely euthymic.

### 4.2 Future Directions

We would encourage further studies of sleep changes before mood episodes in BD in larger samples, but with similarly long study durations. Changes to include objective sleep measures, a more diverse BD sample, that is, not just patients in remission but including clinically more impaired groups, and categorisation of sleep patterns for depressive episodes, that is, tendency for hypersomnia or insomnia, could improve the overall understanding of sleep as a predictor for mood in BD. Given variability changes were our main indicators of increased likelihood of mood episode change, clinicians may consider using digital software or technology to help assist in collecting sleep data and to calculate the variability of sleep, as these calculations may be too complex for patients to do every day. Using EWS in clinical practice has been shown to improve clinical outcomes [2], and our results highlight the use of sleep changes as EWS in patients with BD who are in remission and may therefore not be under intense clinical observation.

## 5 Conclusion

Although having an excellent data set with long study duration and extensive daily data, we did not find changes in mean values of sleep variables to reliably predict either depressive or manic episodes. However, variability in sleep appears to be a promising EWS for predicting mood episodes. In particular, a more unstable pattern of sleep was found before depressive episodes, particularly variability in sleep duration and time waking up. Using variability as EWS could help patients and clinicians prepare for mood changes and make suitable adjustments, something digital technology could be utilised for in future clinical practice. The results are based on a modest sample size. Further exploration of the role of sleep as an EWS for mood episodes in a larger group of BD is warranted.

## Supporting information


**Data S1:** bdi70054‐sup‐0001‐DataS1‐S5.docx.
**Data S2:** bdi70054‐sup‐0001‐DataS1‐S5.docx.
**Data S3:** bdi70054‐sup‐0001‐DataS1‐S5.docx.
**Data S4:** bdi70054‐sup‐0001‐DataS1‐S5.docx.
**Data S5:** bdi70054‐sup‐0001‐DataS1‐S5.docx.

## Data Availability

The data that support the findings of this study are available on request from the corresponding author. The data are not publicly available due to privacy or ethical restrictions.
