# Supplementary material for: Can Sleep Parameters Predict Upcoming Mood Episodes in Bipolar Disorder?
Source: Bipolar Disord. 2025 Aug 22;27(6):449–60. doi: 10.1111/bdi.70054 (PMC12483305; doi:10.1111/bdi.70054)
Supplement: Supplementary file 1 — Data S1: bdi70054‐sup‐0001‐DataS1‐S5.docx. Data S2: bdi70054‐sup‐0001‐DataS1‐S5.docx. Data S3: bdi70054‐sup‐0001‐DataS1‐S5.docx. Data S4: bdi70054‐sup‐0001‐DataS1‐S5.docx. Data S5: bdi70054‐sup‐0001‐DataS1‐S5.docx. [file BDI-27-449-s001.docx]

Data S1: Within_values SPSS syntax

Example SPSS code

Code2=break for each participant to calculate the participant specific value; Sleep_h_total=total hours slept each day; Mean_h_sleep=create new value of the mean hours each participant slept daily over 12 months; within_h_sleep=difference between total hours asleep on specific day for each participant and participant-specific mean over 12 months.

AGGREGATE

/OUTFILE=* MODE=ADDVARIABLES

/BREAK=Code2

/Mean_h_sleep=MEAN(sleep_h_total).

COMPUTE within_h_sleep=sleep_h_total- Mean_h_sleep.

EXECUTE.

Data S2: Multilevel models SPSS syntax

Example SPSS code

Predictor: hours asleep (within_h_sleep); outcome: mood status code for >2 weeks after manic episode (mood2_3wam); filter to only include data that is either for >2 weeks after manic episode or euthymia, filter = 0 or 1, respectively.

USE ALL.

COMPUTE filter_$=((Mood2_3wam = 0) or ( Mood2_3wam = 1)). *This to only include mood status either >2 weeks after manic episode, yes=0 or no=1 respectively.*

FILTER BY filter_$.

EXECUTE.

GENLINMIXED

/DATA_STRUCTURE SUBJECTS=code2 /FIELDS TARGET=mood2_3wam TRIALS=NONE OFFSET=NONE

/TARGET_OPTIONS REFERENCE=1 DISTRIBUTION=MULTINOMIAL LINK=LOGIT

/FIXED EFFECTS=within_h_sleep

USE_INTERCEPT=TRUE

/RANDOM USE_INTERCEPT=TRUE SUBJECTS=Code2 COVARIANCE_TYPE=VARIANCE_COMPONENTS

/BUILD_OPTIONS TARGET_CATEGORY_ORDER=DESCENDING INPUTS_CATEGORY_ORDER=ASCENDING MAX_ITERATIONS=100

CONFIDENCE_LEVEL=95 DF_METHOD=RESIDUAL COVB=MODEL PCONVERGE=0.000001(ABSOLUTE) SCORING=0

SINGULAR=0.000000000001

/EMMEANS_OPTIONS SCALE=ORIGINAL PADJUST=LSD.

Data S3: Bonferroni-Holm Correction

Formular:

$$BH=\frac{Target Alpha}{n-rank+1}$$

Where BH = Bonferroni-Holm Correction

Target Alpha = The chosen level of significance. Target alpha to reach significance levels is 0.05, and to reach high significance levels is 0.01.

N = Number of comparisons included, in our case this is 10.

Rank = what number in order from smallest to largest is the p-value.

For significance to be reached, the original calculated p-value has to be smaller or equal to BH.

| Ranked by smallest p-value | *Episode Phase (ranked)* | *P-value (ranked)* | *Bonferroni-Holm Correction (significance)* | *Bonferroni-Holm Correction (high significance)* |
| --- | --- | --- | --- | --- |
| Sleep duration (hours) |  |  |  |  |
|  | >2 weeks after ME | 0.015 | 0.005 | 0.001 |
|  | 2 weeks before DE | 0.095 | 0.00555556 | 0.001111111 |
|  | 2 weeks after ME | 0.109 | 0.00625 | 0.00125 |
|  | 1 week before DE | 0.479 | 0.00714286 | 0.001428571 |
|  | 1 week before ME | 0.502 | 0.00833333 | 0.001666667 |
|  | >2 weeks after DE | 0.609 | 0.01 | 0.002 |
|  | 2 weeks before ME | 0.785 | 0.0125 | 0.0025 |
|  | 2 weeks after DE | 0.859 | 0.01666667 | 0.003333333 |
|  | 1 week after ME | 0.941 | 0.025 | 0.005 |
|  | 1 week after DE | 0.961 | 0.05 | 0.01 |
| Sleepless in bed (hours) |  |  |  |  |
|  | >2 weeks after DE | 0.023 | 0.005 | 0.001 |
|  | 1 week before ME | 0.03 | 0.00555556 | 0.001111111 |
|  | 2 weeks after ME | 0.033 | 0.00625 | 0.00125 |
|  | 1 week after DE | 0.068 | 0.00714286 | 0.001428571 |
|  | 2 weeks after DE | 0.076 | 0.00833333 | 0.001666667 |
|  | 1 week after ME | 0.183 | 0.01 | 0.002 |
|  | 2 weeks before DE | 0.235 | 0.0125 | 0.0025 |
|  | 2 weeks before ME | 0.482 | 0.01666667 | 0.003333333 |
|  | 1 week before DE | 0.65 | 0.025 | 0.005 |
|  | >2 weeks after ME | 0.769 | 0.05 | 0.01 |
| Time falling asleep |  |  |  |  |
|  | 1 week after DE | 0.01 | 0.005 | 0.001 |
|  | 2 weeks after DE | 0.058 | 0.00555556 | 0.001111111 |
|  | 1 week before ME | 0.277 | 0.00625 | 0.00125 |
|  | >2 weeks after ME | 0.282 | 0.00714286 | 0.001428571 |
|  | 1 week after ME | 0.583 | 0.00833333 | 0.001666667 |
|  | 2 weeks after ME | 0.682 | 0.01 | 0.002 |
|  | 2 weeks before ME | 0.861 | 0.0125 | 0.0025 |
|  | >2 weeks after DE | 0.87 | 0.01666667 | 0.003333333 |
|  | 2 weeks before DE | 0.894 | 0.025 | 0.005 |
|  | 1 week before DE | 0.91 | 0.05 | 0.01 |
| Time waking up |  |  |  |  |
|  | >2 weeks after DE | 0.001** | 0.005 | 0.001 |
|  | 1 week before DE | 0.033 | 0.00555556 | 0.001111111 |
|  | >2 weeks after ME | 0.037 | 0.00625 | 0.00125 |
|  | 1 week before ME | 0.158 | 0.00714286 | 0.001428571 |
|  | 1 week after ME | 0.263 | 0.00833333 | 0.001666667 |
|  | 2 weeks after DE | 0.372 | 0.01 | 0.002 |
|  | 2 weeks before ME | 0.782 | 0.0125 | 0.0025 |
|  | 2 weeks before DE | 0.825 | 0.01666667 | 0.003333333 |
|  | 2 weeks after ME | 0.889 | 0.025 | 0.005 |
|  | 1 week after DE | 0.935 | 0.05 | 0.01 |

DE = Depressive episode. ME = (hypo)mania episode. P = Significance. *= significance after correction for Bonferroni-Holm; **= high significance after correction for Bonferroni-Holm.

| Ranked by smallest p-value | *Episode Phase (ranked)* | *P-value (ranked)* | *Bonferroni-Holm Correction (significance)* | *Bonferroni-Holm Correction (high significance)* |
| --- | --- | --- | --- | --- |
| SD Sleep duration (hours) |  |  |  |  |
|  | 2 weeks before DE | 0.001** | 0.005 | 0.001 |
|  | 1 week before DE | 0.001** | 0.00555556 | 0.001111 |
|  | 1 week after DE | 0.001** | 0.00625 | 0.00125 |
|  | 1 week before ME | 0.007* | 0.00714286 | 0.001429 |
|  | 2 weeks before ME | 0.111 | 0.00833333 | 0.001667 |
|  | >2 weeks after ME | 0.133 | 0.01 | 0.002 |
|  | 2 weeks after DE | 0.196 | 0.0125 | 0.0025 |
|  | >2 weeks after DE | 0.332 | 0.01666667 | 0.003333 |
|  | 1 week after ME | 0.581 | 0.025 | 0.005 |
|  | 2 weeks after ME | 0.904 | 0.05 | 0.01 |
| SD Sleepless in bed (hours) |  |  |  |  |
|  | >2 weeks after DE | 0.001** | 0.005 | 0.001 |
|  | 2 weeks after ME | 0.001** | 0.00555556 | 0.001111 |
|  | 1 week after ME | 0.002* | 0.00625 | 0.00125 |
|  | 2 weeks after DE | 0.011 | 0.00714286 | 0.001429 |
|  | 1 week after DE | 0.021 | 0.00833333 | 0.001667 |
|  | 1 week before ME | 0.031 | 0.01 | 0.002 |
|  | >2 weeks after ME | 0.083 | 0.0125 | 0.0025 |
|  | 1 week before DE | 0.148 | 0.01666667 | 0.003333 |
|  | 2 weeks before ME | 0.269 | 0.025 | 0.005 |
|  | 2 weeks before DE | 0.518 | 0.05 | 0.01 |
| SD Time falling asleep |  |  |  |  |
|  | 1 week before ME | 0.001** | 0.005 | 0.001 |
|  | 2 weeks after ME | 0.026 | 0.00555556 | 0.001111 |
|  | >2 weeks after ME | 0.055 | 0.00625 | 0.00125 |
|  | 1 week after ME | 0.124 | 0.00714286 | 0.001429 |
|  | >2 weeks after DE | 0.18 | 0.00833333 | 0.001667 |
|  | 1 week after DE | 0.369 | 0.01 | 0.002 |
|  | 1 week before DE | 0.394 | 0.0125 | 0.0025 |
|  | 2 weeks after DE | 0.5 | 0.01666667 | 0.003333 |
|  | 2 weeks before ME | 0.879 | 0.025 | 0.005 |
|  | 2 weeks before DE | 0.951 | 0.05 | 0.01 |
| SD Time waking up |  |  |  |  |
|  | 1 week before DE | 0.001** | 0.005 | 0.001 |
|  | 1 week after DE | 0.001** | 0.00555556 | 0.001111 |
|  | 1 week after ME | 0.001** | 0.00625 | 0.00125 |
|  | 1 week before ME | 0.002* | 0.00714286 | 0.001429 |
|  | >2 weeks after ME | 0.003* | 0.00833333 | 0.001667 |
|  | 2 weeks before ME | 0.004* | 0.01 | 0.002 |
|  | 2 weeks after DE | 0.035 | 0.0125 | 0.0025 |
|  | 2 weeks after ME | 0.379 | 0.01666667 | 0.003333 |
|  | >2 weeks after DE | 0.646 | 0.025 | 0.005 |
|  | 2 weeks before DE | 0.857 | 0.05 | 0.01 |

DE = Depressive episode. ME = (hypo)mania episode. P = Significance. *= significance after correction for Bonferroni-Holm; **= high significance after correction for Bonferroni-Holm.

Data S4: Mean hours asleep by episode phase

| Sleep hours | N (episodes) | Minimum | Maximum | Mean | SD |
| --- | --- | --- | --- | --- | --- |
| 2 weeks before DE | 14 | 4.57 | 11.41 | 7.93 | 1.71 |
| 1 week before DE | 16 | 4.36 | 10.85 | 7.86 | 1.63 |
| 1 week after DE | 16 | 4.36 | 10.85 | 7.86 | 1.63 |
| 2 weeks after DE | 16 | 4.67 | 10.20 | 7.71 | 1.65 |
| >2 weeks after DE | 11 | 4.50 | 10.71 | 7.91 | 1.81 |
| 2 weeks before ME episode | 10 | 4.71 | 9.29 | 7.31 | 1.44 |
| 1 week before ME episode | 11 | 5.36 | 9.43 | 7.52 | 1.24 |
| 1 week after ME episode | 11 | 3.79 | 9.79 | 7.33 | 1.96 |
| 2 weeks after ME episode | 11 | 4.50 | 9.83 | 7.20 | 1.66 |
| >2 weeks after ME episode | 5 | 4.54 | 9.25 | 7.02 | 1.93 |
| Euthymic | 29 | 4.82 | 10.13 | 7.87 | 1.24 |

DE=Depressive Episode; ME=(Hypo)Manic Episode; SD=Standard Deviation; N=Number of episodes.

Data S5: Sleep instability results (Sum of squared deviations = SSD)

|  | *Episode Phase* | *Days* | *Coefficient* | *SE* | *P* |
| --- | --- | --- | --- | --- | --- |
| SSD of sleep duration (hours) | Euthymia | 7574-7360 |  |  |  |
|  | 2 weeks before DE | 182 | 0.02 | 0.0062 | 0.001** |
|  | 1 week before DE | 164 | 0.018 | 0.0062 | 0.004** |
|  | 1 week after DE | 182 | 0.018 | 0.006 | 0.003** |
|  | 2 weeks after DE | 191 | 0.009 | 0.0081 | 0.266 |
|  | >2 weeks after DE | 186 | -0.038 | 0.023 | 0.101 |
|  | 2 weeks before ME | 117 | 0.002 | 0.0045 | 0.616 |
|  | 1 week before ME | 104 | -0.081 | 0.036 | 0.025* |
|  | 1 week after ME | 134 | -0.032 | 0.0222 | 0.0151 |
|  | 2 weeks after ME | 139 | -0.041 | 0.0232 | 0.077 |
|  | >2 weeks after ME | 104 | -0.045 | 0.0301 | 0.131 |
| SSD of sleepless in bed (hours) | Euthymia | 7574-7360 |  |  |  |
|  | 2 weeks before DE | 182 | -0.061 | 0.0373 | 0.104 |
|  | 1 week before DE | 164 | 0.034 | 0.011 | 0.002** |
|  | 1 week after DE | 182 | 0.038 | 0.0117 | 0.001** |
|  | 2 weeks after DE | 191 | 0.018 | 0.0147 | 0.224 |
|  | >2 weeks after DE | 186 | -0.002 | 0.0172 | 0.895 |
|  | 2 weeks before ME | 117 | 0.002 | 0.0242 | 0.943 |
|  | 1 week before ME | 104 | 0.028 | 0.0182 | 0.122 |
|  | 1 week after ME | 134 | -0.062 | 0.0377 | 0.098 |
|  | 2 weeks after ME | 139 | -0.202 | 0.0651 | 0.002** |
|  | >2 weeks after ME | 104 | -0.202 | 0.0738 | 0.006** |
| SSD of time falling asleep | Euthymia | 7570-7626 |  |  |  |
|  | 2 weeks before DE | 182 | 0.005 | 0.0167 | 0.746 |
|  | 1 week before DE | 164 | 0.056 | 0.0114 | <0.001** |
|  | 1 week after DE | 178 | 0.054 | 0.01 | <0.001** |
|  | 2 weeks after DE | 187 | 0.034 | 0.0091 | <0.001** |
|  | >2 weeks after DE | 186 | -0.06 | 0.0239 | 0.012* |
|  | 2 weeks before ME | 117 | -0.108 | 0.0413 | 0.009* |
|  | 1 week before ME | 104 | 0.01 | 0.0243 | 0.695 |
|  | 1 week after ME | 134 | -0.179 | 0.0455 | <0.001** |
|  | 2 weeks after ME | 139 | -0.176 | 0.0438 | <0.001** |
|  | >2 weeks after ME | 104 | -0.117 | 0.0446 | 0.009* |
| SSD of time waking up | Euthymia | 7492-7548 |  |  |  |
|  | 2 weeks before DE | 182 | -0.008 | 0.0456 | 0.863 |
|  | 1 week before DE | 166 | -0.075 | 0.055 | 0.174 |
|  | 1 week after DE | 204 | 0.012 | 0.0409 | 0.774 |
|  | 2 weeks after DE | 191 | -0.037 | 0.0486 | 0.443 |
|  | >2 weeks after DE | 186 | -0.075 | 0.0457 | 0.101 |
|  | 2 weeks before ME | 122 | 0.006 | 0.0388 | 0.88 |
|  | 1 week before ME | 112 | -0.079 | 0.0473 | 0.094 |
|  | 1 week after ME | 140 | 0.004 | 0.0359 | 0.912 |
|  | 2 weeks after ME | 139 | -0.085 | 0.0432 | 0.05* |
|  | >2 weeks after ME | 104 | 0.02 | 0.493 | 0.662 |

DE = Depressive episode. ME = (hypo)mania episode. SSD = sum of squared deviations. SE = Standard Error. P = Significance. *= 5% significance or less; **= 1% significance or less.
